# Microvascular anatomy of the brain of the adult pipid frog, *Xenopus laevis* (Daudin): A scanning electron microscopic study of vascular corrosion casts

**DOI:** 10.1002/jmor.20824

**Published:** 2018-04-25

**Authors:** Alois Lametschwandtner, Bernd Minnich

**Affiliations:** ^1^ Department of Biosciences, Vascular and Performance Biology Research Group, Hellbrunnerstrasse 34 University of Salzburg Salzburg Austria

**Keywords:** CNS, choroid plexuses, histomorphology, spinal cord, Willis circle

## Abstract

To demonstrate the 3D microvascular anatomy of the brain of the model organism *Xenopus laevis* Daudin scanning electron microscopy of vascular corrosion casts was correlated with light microscopy of stained 7 µm thick serial tissues sections. Results showed that supplying arteries descended from the leptomeningeal surface without remarkable branchings straight to the subventricular zone where they branched and capillarized. Capillaries showed few H‐ and/or Y‐shaped anastomoses during their centrifugal course toward the leptomeningeal surface where they drained into cerebral venules and veins. Apart from the accessory olfactory bulb and the vestibule‐cochlear nucleus where capillaries were densely packed, capillaries formed a wide‐meshed 3D network throughout the brain parenchyma and thus contrasted to urodelian brains where hairpin‐shaped capillaries descend from the leptomeningeal vessels into varying depths of the brain parenchyma. In about two‐third of specimens, a closed arterial circle of Willis was found at the base of the brain. If this circle in *Xenopus* might serve the same two functions as in men is briefly discussed. Choroid plexuses of third and fourth ventricle were found to have a high venous, but a low arterial inflow via one small choroidal artery only. Findings are compared with previous studies on the vascularization of the anuran brain and discrepancies in respect to presence or absence of particular arteries and/or veins in Ranids, Bufonids, and Pipids studied so far are discussed with particular emphasis on the techniques used in the various studies published so far.

**Abbreviations**
aa: auditory artery
ab: anterior branch of cerebral carotid artery
acn: accumbens nucleus
aima: anterior inferior mesencephalic artery
alhy: anterior lobe of hypophysis
aob: accessory olfactory bulb
asma: anterior superior mesencephalic artery
ba: basilar artery
bltv: branch of lateral telencephalic vein
c: capillary
ca: central artery
cava: communicating artery with vertebral artery
cca: cerebral carotid artery
cer: cerebellum
cf: choroidal fold
cha: choroidal artery
cht: choroidal tela
cp III: choroid plexus of the third ventricle
cp IV: choroid plexus of fourth ventricle
cv: choroidal vein
cvr: cochlear‐vestibular nuclear region
da: diencephalic artery
di: diencephalon
dp: dorsal pallium
dv: diencephalic vein
dia: deep infundibular artery
dsv: dorsal spinal vein
dth: dorsal thalamus
epi: epithalamus
eppv: encephalo‐posthypophyseal portal vein
fom: foramen of Monroe
hb: hypothalamic branch of encephalo‐posthypophyseal portal vein
hy: hypopyhsis
hyp: hypothalamus
hyv: hypophyseal vein
hv: hypothalamic vein
ihv: interhemispheric vein
ilhy: intermediate lobe of hypophysis
ir: infundibular recess
lamv: lateral mesencephalic vein
ldv: lateral diencephalic vein
lmv: longitudinal mesencephalic vein
loa: lateral olfactory artery
lot: lateral optic tectum
lp: lateral pallium
ltv: lateral telencephalic vein
lv: lateral ventricle
ma: meningeal artery
me: median eminence
mes: mesencephalon
moa: medial olfactory artery
mp: medial pallium
mv: mesencephalic ventricle
och: optic chiasm
ocv: oblique cranial vein
oov: oblique occipital vein
ot: optic tectum
ov: ophthalmic vein
pa: preoptic area
pal: pallium
par: paraphysis
pb: posterior branch of cerebral carotid artery
pcv: prootic cranial vein
plhy: posterior lobe of hypophysis
poa: preoptic artery
psma: posterior superior mesencephalic artery
pta: posterior telencephalic artery
rcv: retrochiasmatic vein
rf: rhombencephalic fossa
rho: rhombencephalon
rica: retroinfundibular communicating artery
rot: rostral optic tectum
rta: rostral tegmental artery
sa: striatal artery
se: septum
sia: superficial infundibular artery
st: semicircular torus
str: striatum
sv: sagittal vein
teg: tegmentum
tel: telencephalon
th: thalamus
tv: tegmental vein
v: vein
vIII: third ventricle
vIV: fourth ventricle
va: vertebral artery
vth: ventral thalamus
volt: vascular organ of the lamina terminalis
vtb: ventral tegmental branch of the encephalo‐posthypophyseal portal vein
vtv: ventral tegmental vein
vv: ventral venule

## 1 INTRODUCTION

The vertebrate brain cannot store energy and thus relies on a permanent supply with nutrients and oxygen via the circulatory system. Presently, we have a detailed knowledge of anatomy and efferent and afferent neural connections of the functional systems of the anuran brain (for reviews see e.g., Kemali & Braitenberg, 1969; Kuhlenbeck, 1977; Llinas & Precht, 1976; Ten Donkelaar, 1998). Comparatively little, however, is known on its detailed microvascular anatomy. Studies mainly focus on gross arterial supply and venous drainage (Abbie, 1934; Craigie, 1938; Gaupp, 1899; Gillilan, 1967; Millard, 1940, 1945, 1949; Schöbl, 1882; Socha, 1930) or describe the vascularization of specific brain areas only (Cruz, 1959; Dierickx, Goossens, & De Waele, 1970, 1974; Dierickx, Lombaerts‐Vandenberghe, & Druyts, 1971; Goossens, Dierickx, & De Waele, 1973; Rodriguez & Pizzi, 1967). In most studies, authors use India‐ink or India ink‐gelatin injections with subsequent clearing of the brain tissue according to Spalteholz (1914) and study these specimens either in toto using the stereomicroscope or analyze serially thick‐sectioned brains by conventional light microscopy (LM). In India‐ink injected specimens, it is difficult to positively differentiate arteries from veins and vessels have to be followed from their identifiable origin. Though differentiation of vessel nature can be improved by the double injection technique (Ambach & Palkovits, 1974), the low depth of focus of the light microscope requires amendment by serial sectioning and laborious reconstruction work if information about the entire cerebrovascular system is needed. Staining of vessels by perfusion with lipophilic DiI (vessel painting; Hughes, Dashkin, & Defazio, 2014) followed by observation with conventional fluorescence microscopy or confocal microscopy improves spatial resolution and depth of focus, but due to the limited depth of penetration of the laser this method still relies on thick sectioning of larger brains.

The availability of resins which enable to cast the entire vascular bed from the aortic trunk(s) through the capillaries to the opened heart (Taniguchi, Ohta, & Tajiri, 1952) and the application of the scanning electron microscope (SEM) to study these resin‐made vascular casts (Murakami, 1971) enable to document the 3D arrangement of blood vessels with a high depth of focus and a high spatial resolution. The possibility to differentiate arterial and venous vessels by means of their characteristic endothelial cell nuclear imprints on cast surfaces (Miodonski, Hodde, & Bakker, 1976) and the application of 3D morphometry (Malkusch, Konerding, Klapthor, & Bruch, 1995; Minnich, Leeb, Bernroider, & Lametschwandtner, 1999) even allow to gain quantitative data on physiologically relevant vascular parameters like vessel diameters, lengths, and branching angles (Stöttinger, Klein, Minnich, & Lametschwandtner, 2006).

Studies of the 3D microvascular anatomy of the whole amphibian brain or parts thereof are still few and deal with three anuran species (*Bufo bufo*: Albrecht, Lametschwandtner, & Adam, 1978, 1980a, 1980b; Lametschwandtner & Simonsberger, 1975; Lametschwandtner, Albrecht, & Adam, 1979a, 1979b; Lametschwandtner, Simonsberger, & Adam, 1976, 1977a, 1977b, 1977c; *Rana pipiens; Rana catesbeiana*: Hinton, Nelson, & Gattone, 1990), and two urodelian species only (*Triturus cristatus and Triturus carnifex*: Lazzari, Ciani, & Franceschini, 1991; *Ambystoma mexicanum*: Lazzari & Franceschini, 2003).

To date, we have a 3D, high‐resolution atlas of normal vascular development in the embryo (Levine, Munoz‐Sanjuan, Bell, North, & Brivanlou, 2003) and some knowledge of the angiogenesis within the optic tectum of the embryo, tadpole, and postmetamorphic *Xenopus laevis* (Rovainen & Kakarala, 1989; Tiedeken & Rovainen, 1991). However, we still lack an in‐depth knowledge of the brain's 3D microvascular anatomy of this model organism in biological research. A profound knowledge of microvascular patterns and in particular of vascular connections is crucial to better understand energy supply and distribution within neuroanatomically clearly defined brain areas. Here, we demonstrate that SEM of vascular corrosion casts in combination with LM of stained serial tissue sections enables to visualize minute details of the microvascular bed and to topologically attribute them to distinct small brain areas. Additionally, we show that color‐coding of arteries (red), veins (blue), and meningeal vessels (green) in SEM micrographs distinctly facilitates identification of vessel origins, courses, branching patterns, and areas of supply and drainage.

## 2 MATERIALS AND METHODS

### 2.1 Animals

Ten adult animals (four males, 34–43 g, body length: 7.5–8.0 cm; six females; 33–100 g, body length: 6.3–10.0 cm) of the pipid frog, *X. laevis* (Daudin) were studied. The larger animals were purchased from Horst Kaehler (Hamburg, Germany), the smaller ones were raised in our animal facility. Animals were housed in aquaria (tap water; depth: 15 cm) equipped with aquarium filters and fed twice a week with either dried Gammarus pulex or grinded beef heart.

### 2.2 Histomorphology

One male (body weight: 77.0 g, body length: 8.0 cm) and one female animal (body weight: 78 g, body length: 10.0 cm) were killed by immersion into an aqueous solution of MS 222 (0.5%; Sigma‐Aldrich Chemie, Steinbuch, Germany). After, weighing animals were pinned in supine position on a wax plate. The heart with bulbus cordis and truncus arteriosus was exposed by thoracotomy and a ligature was placed around the bulbus cordis. Next, the ventricle was cut open and a blunt grinded vein flow G19 (Braun, Melsungen, Germany) guided by a micromanipulator was inserted through the opened ventricle into the truncus arteriosus. Subsequently, the blunted needle was tied in place with a ligature from thread to ensure its stability during the following rinsing and fixing processes. Finally, the sinus venosus was cut open to allow efflux of blood and rinsing with amphibian ringer solution (Adam & Czihak, 1964) started. The flow rate of the infusion pump (Habel, Vienna) was set to 40 mL/hr. When clear reflux drained from the opened sinus venosus fixation with 10 mL Bouin's solution (Adam & Czihak, 1964) was started using the same flow rate. The fixed brain was removed from the brain cavity, dehydrated, and embedded in paraplast. One series each of 7 µm thick transverse and of longitudinal sections were stained according to Goldner (Adam & Czihak, 1964). Tissue sections were analyzed with an Olympus X51 microscope. Images were recorded by a Color View III digital camera (Soft Imaging Systems, FRG). If necessary brightness and contrast of images were adjusted using Photoshop 7.0 (Adobe Inc., Redwood, CA).

### 2.3 Vascular corrosion casting

Seventeen adult *X. laevis* (4 males, 13 females; body weights: 39.0–93.0 g, total lengths: 75–95 mm) were studied. For euthanasia and rinsing, see Section 2.2. When clear reflux drained from the opened sinus venosus 10 ml of Mercox CL‐2B (Dainippon Ink and Chemicals, Tokyo, Japan; Ladd Burlington, Vermont, USA) diluted with monomeric methyl methacrylate (4 + 1, v + v, 10 mL monomeric methyl acrylate contained 0.85 g initiator paste MA) were injected with the infusor (see Section 2.2) at a flow rate of 41 mL/hr. When the effluent resin became viscous (after 13–14 min) or the whole amount of resin had been perfused the injection was stopped and the animals were left for about 30 min at room temperature to allow hardening of the injected resin. Animals then were put into a water‐bath (60°C; 12–24 hr) to temper the injected resin. Next, specimens were macerated in potassium hydroxide (7.5%; 40°C; 2–24 hr), rinsed three times in distilled water, submerged in 2% hydrochloric acid, rinsed three times in distilled water followed by submersion in formic acid (5%; 20°C; 5–15 min) to dissolve any residual organic matter adhering to the cast surfaces. Finally, specimens were rinsed another three times in distilled water and frozen in fresh distilled water. Ice‐embedded casts were freeze‐dried in a Lyovac GT2 (Leybold‐Heraeus, Cologne, Germany). Brains were excised and mounted onto specimen stubs using the “conductive bridge‐method” (Lametschwandtner, Miodonski, & Simonsberger, 1980), either evaporated with carbon and gold and/or sputter‐coated with gold, and examined in the SEM ESEM XL‐30 (FEI, Eindhoven, The Netherlands) at an accelerating voltage of 10 kV.

Individual cast brains were either mounted in toto onto specimen stubs for detailed SEM analyses or were cut transversely or sagittally. For this purpose, brain casts were submerged into distilled water, frozen and sectioned while embedded in ice using a mini‐wheel saw placed in the cryo‐chamber of a cryo‐microtome (Lametschwandtner & Lametschwandtner, 1992). Sectioned casts where cleaned, frozen, freeze‐dried, mounted, and analyzed in the SEM.

In some specimen's course, branching patterns and areas of supply (or drainage) of individual vessels were exposed by ripping‐off overlaying vessels under binocular control by fine tipped insect pins.

## 3 RESULTS

Stereomicroscopic inspection of whole brain vascular corrosion casts revealed excellent filling of pial vessels. Subsequent SEM of sectioned or partially dissected casts confirmed that also intraparenchymal microvessels were fully replicated and imposed as a 3D network whose density was highest in the accessory olfactory bulb and the vestibulo‐cochlear nucleus, but low in the remaining brain areas.

To unravel the 3D vascular network of the entire brain and spinal cord, we first report gross arterial supply. Starting with extra‐ and intracranial feeding vessels, we follow their branches intraparenchymally toward the microvascular bed. Next, we trace gross venous drainage routes toward the pial surface and finally analyze microvascular patterns.

### 3.1 Gross arterial supply

**Figure 1:**
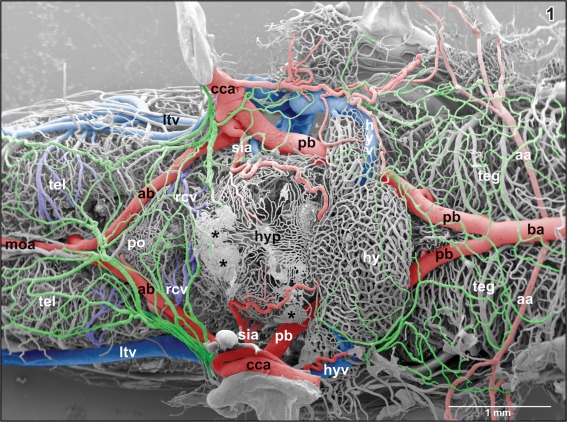
Microvascular anatomy of the brain in adult *X. laevis*. Vascular corrosion cast. SEM. Ventral view displaying telencephalic hemispheres (tel), preoptic area (pa), hypothalamus (hyp), hypophysis (hy), and mesencephalic and rhombencephalic tegmentum (teg). Arterial vessels are red, venous vessels are blue, and meningeal vessels are green. Asterisks mark extravasates. For further abbreviations in this and following figures see the list of abbreviations

**Figure 2:**
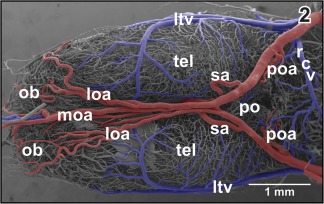
Gross arterial supply and venous drainage of olfactory bulb (ob), telencephalon (tel), and preoptic area (pa). Ventral view

**Figure 3:**
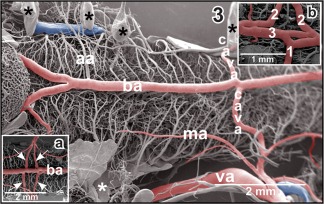
Anatomy of the vertebro‐basilar arterial system. **Inset a**. Communicating arteries (arrows) bifurcate before they join the basilar artery (ba) bilaterally. **Inset b**. Asymmetric junctions of left (1) and right (2) communicating arteries with the basilar artery (3). Asterisks mark conductive bridges

The brain of adult *X. laevis* is bilaterally fed via (a) common carotid artery—internal carotid artery—cerebral carotid artery and (b) the vertebral artery which connects via the communicating artery with the single basilar artery (Figures 1, 2, 3). In general, caliber (luminal diameter), origin and course of branches of anterior and posterior branches of the cerebral carotid artery may vary greatly inter‐ and intraindividually between left and right sides.

The cerebral carotid artery enters the cranial cavity through the prootic foramen and then bifurcates into an anterior branch and a posterior branch (Figure 1). The anterior branch of the cerebral carotid artery—termed ramus hemispherii medialis ventralis by Dierickx et al. (1974) in *Rana temporaria*—courses rostromedially along the ventral border between telencephalon and diencephalon toward the caudal end of the ventral interhemispheric fissure. Here, right and left branches join in most specimens and establish the anterior closure of the arterial circle of Willis (Figure 2).

**Figure 4:**
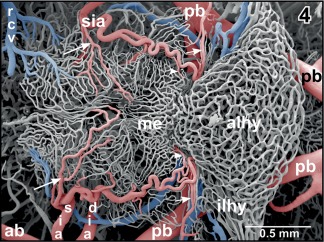
Microvascular anatomy of the ventral hypothalamo‐hypophysial region. Ventral aspect. Note the superficial infundibular artery (sia) which gives off a medially directed branch (large arrows) supplying the delicate capillary network of the retrochiasmatic (rostral) and infundibular (caudal) region. The parent artery bends toward caudal to supply the median eminence (me) via a medially directed branch (arrowheads) and the intermediate lobe of the hypophysis (ilhy) via a laterally directed terminal branch (small arrows)

**Figure 5:**
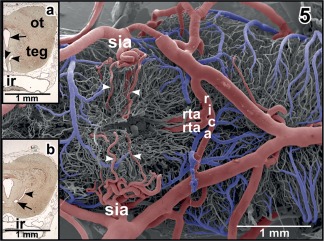
Same as Figure 4 but after removal of vascular beds of infundibular region (ventral hypothalamus) and hypophysis. Note the deeply penetrating branches of the superficial infundibular arteries (sia; arrowheads), the retroinfundibular communicating artery (rica) and the origin of prominent ascending rostral tegmental arteries (rta). **Inset a**. Histomorphology of (right) optic tectum (ot) and tegmentum (teg) of the mesencephalon. Paraplast embedded Goldner stained tissue section (7 µm). Transverse section at the level of the ascending rostral tegmental arteries (arrowheads). Arrow marks the mesencephalic ventricle. ir, infundibular recess. **Inset b**. Same as **inset a**, but slightly more caudal section. Note the horizontally running caudal branch of the (right) rostral tegmental artery (arrow) and its ascending branch (arrowhead)

**Figure 6:**
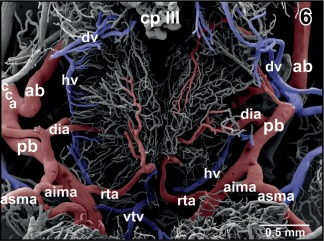
Arterial supply and venous drainage of the dorsal hypothalamus. Note the arterial supply via the deep infundibular artery (dia) and subependymally located rostral branches of rostral tegmental arteries (rta). The diencephalic vein (dv) drains rostral areas while the hypothalamic vein (hv) drains caudal areas

**Figure 7:**
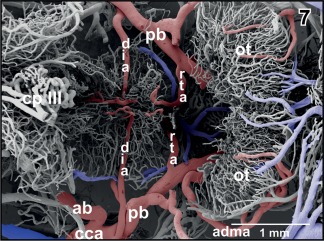
Same specimen as in Figure 6, but after exposure of the deep infundibular artery by removal of overlaying vessels. Note that the artery runs without branching straight toward the subependymal zone where it branches into rostrally and caudally directed branches

The posterior branch of the cerebral carotid artery first extends along the lateral border between dorsal hypothalamus and mesencephalic tegmentum toward caudal. At about the level of the anterior margin of the distal lobe of the hypophysis, it branches off the retroinfundibular communicating artery which connects transversely with the opposite fellow vessel (Figures 4, 5, 6, 7). Slightly behind the caudal margin of the hypophysis, right and left posterior branches unite in the midline of the ventral surface of the mesencephalic tegmentum and continue toward caudally as medially located (single) basilar artery (Figures 1, 3, and 5). About 3–4 mm caudally to this junction, the basilar artery is bilaterally joined by an artery which connects it with the vertebral arteries. These vessels are termed communicating arteries with the vertebral arteries (Figure 3). The basilar artery continues as ventral spinal artery toward the caudal end of the spinal cord (Figure 3).

Anterior branches of the cerebral carotid artery on their course toward rostral issue the following branches (Figures 1 and 2):

1. Superficial infundibular artery,
2. Deep infundibular artery,
3. Preoptic artery,
4. Septal artery,
5. Posterior telencephalic artery (also termed ramus hemispherii medialis dorsalis by Dierickx et al., 1970), and
6. Olfactory arteries (medial, lateral).

The **superficial infundibular artery** either arises with a common stem together with the deep infundibular artery (Figure 5) or alone, whereby right and left sides may differ in this respect. The superficial infundibular artery first runs ventro‐medially and issues a rostro‐medially directed branch, that is, the **retrochiasmatic artery** (Figure 4). The parent vessel then bends toward caudally and extends along the lateral surface of the infundibular lobe (ventral hypothalamus) toward the lateral median eminence (Figures 1 and 4). On its course, the artery issues side branches which either descend into the depth of the ventral hypothalamus (Figures 4 and 5) or form a single layered superficial capillary network at the transition from the caudal infundibular lobe into the rostral median eminence (Figures 1 and 4). The main trunk continues caudally to finally supply caudal median eminence and intermediate lobe of the hypophysis (Figure 4).

The **deep infundibular artery** runs horizontally toward the subependymal region of the dorsal hypothalamus (Figures 1, 4, 6, and 7). Here, it branches into rostrally, laterally, and caudally directed branches which supply the infundibular lobe in a centrifugal manner (Figure 7).

**Figure 8:**
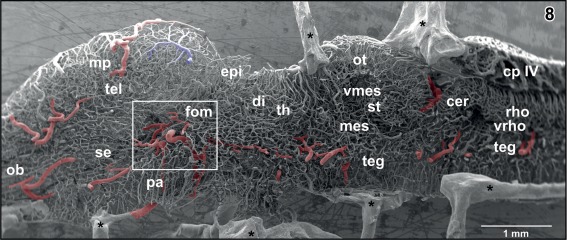
Internal microvascular anatomy of the brain of adult *X. laevis*. Sagittally sectioned vascular corrosion cast. Right half of the brain displaying telencephalon (tel), diencephalon (di) (without hypothalamus and hypophysis), mesencephalon (mes), cerebellum (cer), and rhombencephalon (rho). Asterisks mark conductive bridges

**Figure 9:**
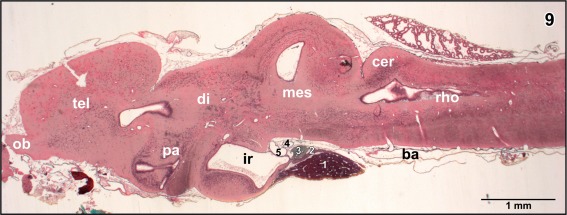
Histomorphology of the brain of adult *X. laevis*. Paraplast embedding. Goldner stained sagittal section (7 µm) at a similar level as displayed in Figure 8. (1) anterior lobe of hypophysis, (2) intermediate lobe of hypophysis, (3) posterior lobe of hypophysis, (4) retroinfundibular communicating artery, and (5) encephalo‐posthypophysial portal vein

**Figure 10:**
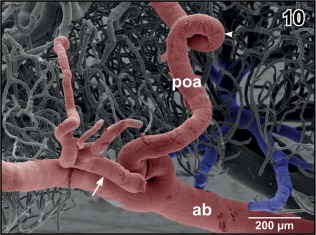
Origin of the preoptic artery (poa) from the anterior branch of the cerebral carotid artery (ab). Note the coiling of the artery (arrowhead) and the side‐branch of the artery (arrow)

**Figure 11:**
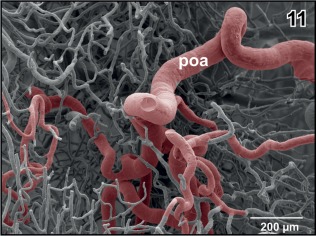
Branching pattern of the preoptic artery (poa) within the subependymal zone of the preoptic area. Note the branches directed anteriorly, laterally, and caudally. Anterior is to the left

The **preoptic artery** arises either from the medial, dorsal, or lateral aspect of the anterior branch of the cerebral carotid artery. It supplies part of the preoptic area and caudal pallial and septal areas of the telencephalon (Figures 1, 8, 9, 10, and 11).

**Figure 12:**
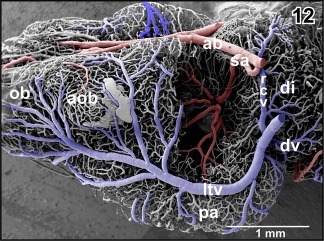
Exposed terminal branching of the striatal artery (sa). Lateral view

**Figure 13:**
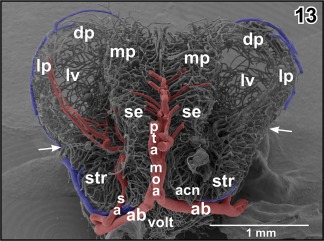
Microvascular anatomy of pallial, septal, and striatal areas. Transverse section. Oblique frontal view. Arrows mark external border between pallium and subpallium

**Figure 14:**
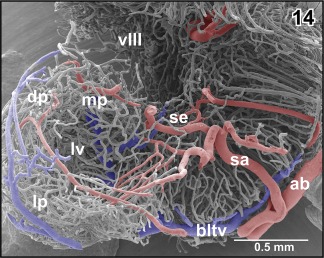
Course and subependymal branching of the striatal artery (sa). Fronto‐lateral view

A prominent **septal artery** was found in a few specimens only (Figures 2 and 12−14). Generally, this artery ascends without any branching through the striatum toward the subventricular zone where it branches intensively (Figures 12, 13, 14). Branches supply striatal, pallial, and septal areas (Figures 12, 13, 14).

**Figure 15:**
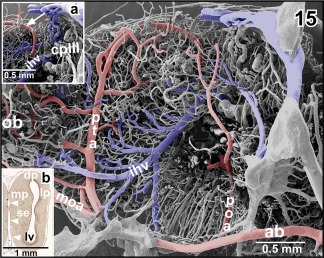
Origin, course, and areas of supply of the posterior telencephalic artery (pta). **Inset a**. Terminal portion of the posterior telencephalic artery (arrow). **Inset b**. Histomorphology of the right telencephalic hemisphere. Transverse section (7 µm). Goldner staining. Note the ascending posterior telencephalic artery (arrowheads)

**Figure 16:**
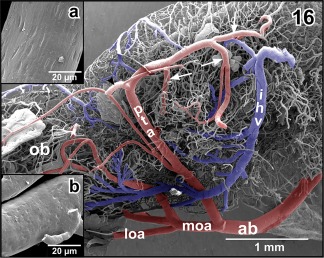
Obliquely ascending posterior telencephalic artery (pta) giving off a rostrally directed branch at the level of the dorsomedial pallium (arrowhead). The main trunk bends toward caudal and issues several branches (arrows). **Inset a**. Characteristic endothelial cell nuclei imprints (arrowheads) at the surface of a cast artery (posterior telencephalic artery). Imprints are longish and orientate parallel to the vessel axis. **Inset b**. Characteristic endothelial cell nuclei imprints (arrowheads) at the surface of a cast vein (interhemispheric vein). Imprints are oval to roundish and orientate randomly

**Figure 17:**
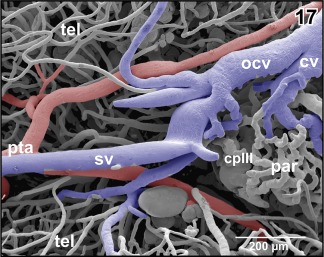
Bifurcation of the posterior telencephalic artery (pta) at the border area between medio‐caudal poles of telencephalic hemispheres (tel) and choroid plexus of the third ventricle (cp III)

In most specimens studied, right and left anterior branches of the cerebral carotid arteries join at about the caudal end of the ventral interhemispheric fissure (Figures 1 and 2). At this site or slightly anteriorly or posteriorly **medial and lateral olfactory arteries** and the **posterior telencephalic artery** arise. While the former run rostrally to supply olfactory and accessory olfactory bulbs, the latter ascends between the telencephalic hemispheres toward the dorsal interhemispheric fissure (Figures 15 and 16). The artery either ascends vertically (Figure 15; see also inset b) or ascends slightly obliquely in a rostral direction (Figure 16). On its course, the artery gives off rostral branches which supply rostral telencephalic areas and caudal areas of the olfactory bulbs (Figure 15). Its caudally directed branches supply septal and medial pallial areas (Figures 15, inset a. and 16). Upon arrival at the dorsal interhemispheric fissure, the posterior telencephalic artery bifurcates into rostrally and caudally directed branches (Figure 16). The former branch supplies the meninges overlaying the rostro‐dorsal telencephalic hemispheres, while the latter supplies medial and dorsal pallial areas of the caudal poles of the telencephalon (Figures 15, 16, 17).

The posterior branch of the cerebral carotid artery issues the following arteries (from rostral to caudal):

1. Deep infundibular artery (in some specimens only, see above),
2. Diencephalic artery,
3. Anterior inferior and superior mesencephalic arteries,
4. Retroinfundibular communicating artery,
5. Posterior superior mesencephalic artery, and
6. Tegmental arteries.

For origin, course, branching pattern, and areas of supply of the **deep infundibular artery**, see above.

**Figure 18:**
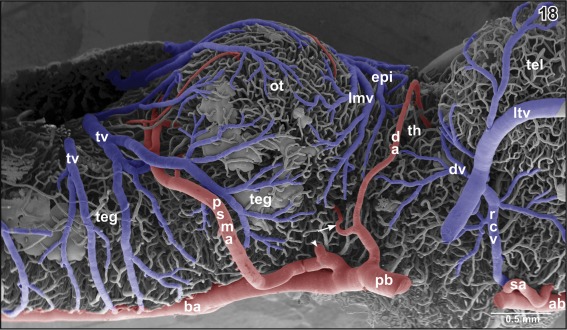
Microvascular anatomy of the pial surface of the posterior pole of the telencephalon (tel), epithalamus (epi), thalamus (th), optic tectum (ot), mesencephalic, and rhombencephalic tegmentum (teg). Lateral view. Rostral is to the right. Note the small caliber of the diencephalic artery (da) which shares a common stem with a lateral tegmental artery (arrow). The posterior superior mesencephalic artery (psma) shares a common stem with the inferior mesencephalic (tegmental) artery (arrowhead)

**Figure 19:**
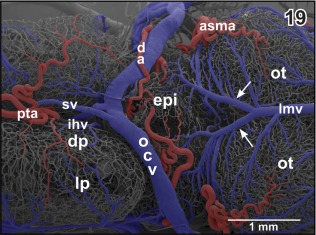
Microvascular anatomy of caudal dorsal (dp) and lateral pallium (lp), epithalamus (epi) and optic tectum (ot). Dorso‐lateral view. Note the prominent oblique cranial vein (ocv) and the medially located longitudinal mesencephalic vein (lmv) which by two tributaries (arrows) drains epithalamic, thalamic and rostral, and lateral areas of the optic tecta

**Figure 20:**
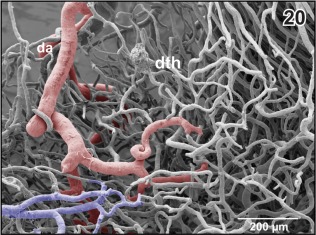
Branch of the diencephalic artery (da) within the (right) dorsal thalamus (dth) after removal of subependymal vessels. Ventricular view. Rostral is to the left

**Figure 21:**
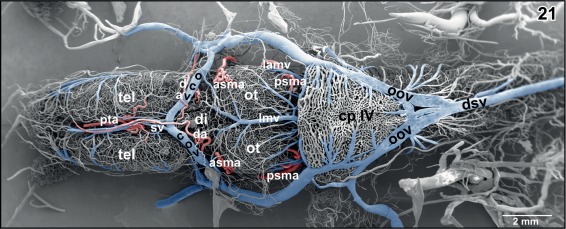
Microvascular anatomy of the brain of adult *X. laevis*. Dorsal view. Note the dorsal location of cranial veins

The **diencephalic artery** either arises individually or with a common stem together with the **anterior inferior mesencephalic artery** (Figure 18). It ascends along the lateral surface of the diencephalon and gives off a first branch which supplies the thalamus (Figures 19 and 20). Next, it issues branches which supply the epithalamus and the epiphysis (Figure 19). The diencephalic artery then courses toward rostro‐medially to finally supply parts of the meninges overlaying the dorso‐caudal telencephalic hemispheres (Figure 21). The right or the left diencephalic artery only issues the **choroidal artery** which feeds the paraphysis and choroid plexus of the third ventricle (Figure 19).

**Figure 22:**
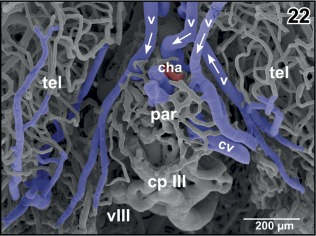
Vascular patterns of paraphysis (par) and choroid plexus of the third ventricle (cp III). Dorsal aspect. Note the veins (v) draining medial pallial areas into the choroid plexus. Arrows indicate proposed direction of blood flow

The **paraphysis** owns a small network of capillary‐sized vessels which either drain into venules coming from the caudal poles of the telencephalon and drain into the oblique cerebral vein or interconnect with the wide‐sinusoid vessels of the choroid plexus of the third ventricle (Figures 17 and 22).

**Figure 23:**
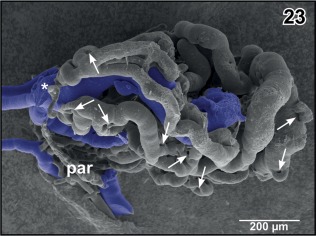
Isolated choroid plexus of the third ventricle. Same specimen as in Figure 22, but ventro‐lateral aspect. The interhemispheric vein (asterisk) continues as wide sinusoid within the plexus. Note the many sites of ongoing intussusceptive microvascular growth (arrows)

The **choroid plexus** of the third ventricle receives its main inflow by the interhemispheric vein which approaches the plexus at its rostroventral border (Figures 15, inset a and 23, asterisk). Additionally, veins from medial and caudal pallial areas feed the plexus (Figure 22). The interhemispheric vein branches into wide‐sinusoid vessels which interconnect by smaller‐sized sinusoids (Figure 23). At many sites, holes of varying shapes and sizes indicate ongoing intussusceptive angiogenesis (Figure 23, arrows).

**Figure 24:**
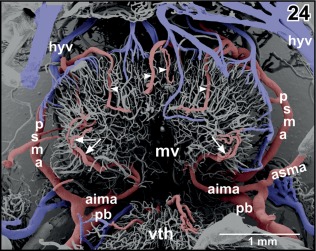
Arterial supply and venous drainage of the mesencephalon (parts of the rostral optic tecta are removed). Anterior is at bottom. Note the prominent anterior inferior mesencephalic arteries (aima) which first penetrate deeply into subependymal areas and then curve in a semicircular manner (arrows). Branches from the posterior superior mesencephalic arteries (psma) radiate from caudally deep into the optic tecta (arrowheads)

**Figure 25:**
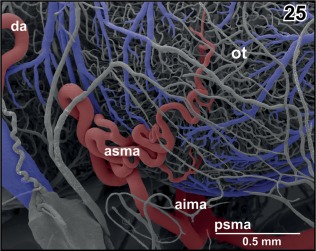
Origin and course of anterior superior mesencephalic artery (asma). Note the coiling of the artery at the rostrolateral area of the optic tectum (ot). Rostral is to the left

**Figure 26:**
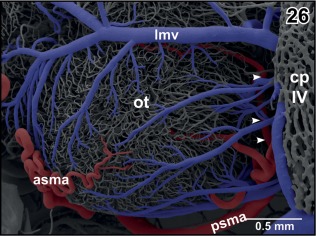
Arterial supply and venous drainage of the left optic tectum. Rostral is to the left. Note the transverse course of the posterior superior mesencephalic artery (psma; arrowheads) within the cleft between optic tectum (ot) and cerebellum (hidden by the overlying choroid plexus of the fourth ventricle (cp IV)

**Figure 27:**
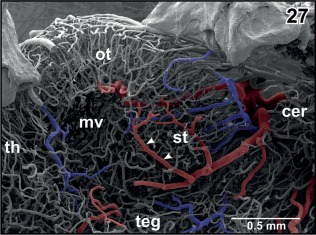
Arterial supply and venous drainage of the semicircular torus (st). Note the location of the innermost arteriole (arrowheads) close beneath the subependymal capillary bed and the radial arrangement of tectal vessels

**Figure 28:**
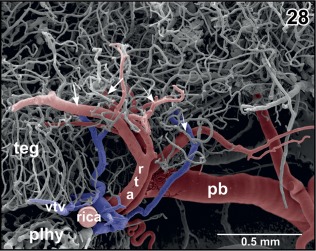
Origin of the rostral tegmental arteries (rta) from the retroinfundibular communicating artery (rica). Lateral view. Note the bifurcations of the arteries into anteriorly (small arrows) and posteriorly directed branches (large arrows)

**Figure 29:**
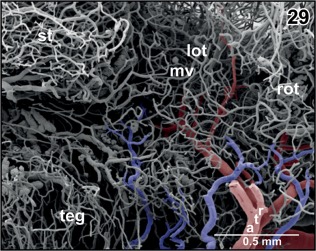
Course and branching pattern of branches of rostral tegmental arteries (rta). Note that caudal branches terminate in the lateral optic tectum (lot) and rostral branches in the rostral optic tectum (rot)

In most specimens, the mesencephalon bilaterally owns one **inferior** and two **superior mesencephalic arteries** (Figures 5, 6, 7, 18, 19, 21, 24, 25, 26, 27, 28, 29, and 31) and several **tegmental arteries** (Figures 28 and 29). The **anterior inferior mesencephalic artery**—which also could be termed a tegmental artery—arises either from the diencephalic artery (Figure 18) or from the anterior superior mesencephalic artery (Figure 25). The anterior inferior mesencephalic artery pierces the latero‐ventral tegmental surface und runs without branching horizontally toward the midline. Here, it ascends vertically, bends toward laterally and runs subependymally to about half height of the optic tectum (Figure 24). The artery supplies the subependymal capillary network and the layers of the optic tectum in a centrifugal manner (Figure 24).

In most specimens, a **retroinfundibular communicating artery** connects right and left posterior branches of the cerebral carotid arteries (Figures 4, 5, 6, 7). The caliber of this artery often varies greatly between its right and left sided portion. In few cases, the middle portion of this artery is very thin or is even absent and right and left stems of the artery do not interconnect at the midline (Figures 6 and 7). In this case, these arteries directly continue as the most rostral (mesencephalic) **tegmental arteries** (Figures 6 and 7). Tegmental arteries ascend in a rostro‐ or caudo‐dorsal direction. In general, they bifurcate into a rostrally and caudally directed branch. The rostral branch supplies caudal areas of dorsal hypothalamus and thalamus (Figures 28 and 29), the caudal branch supplies the mesencephalic tegmentum and ventral and lateral areas of the optic tectum (Figure 29).

The **anterior superior mesencephalic artery** branches off the proximal portion of the caudal superior mesencephalic artery (Figure 25). While ascending along the rostro‐lateral area of the optic tectum, it shows conspicuous coilings and branchings (Figures 19, 21, 25, and 26). Terminal branches finally pierce the surface of the optic tectum (Figure 25).

The **posterior superior mesencephalic artery** first ascends in a dorso‐caudal direction along the lateral surface of the mesencephalic tegmentum (Figures 18, 21, 24, 25, 26). At the border region to the cerebellum, it bends toward medially and issues branches which pierce the pial surface in an acute angle (Figures 24 and 27). The main trunk continues in the cleft between optic tectum and anterior surface of the cerebellum toward the midline whereby it issues further branches. Branches reach centripetally toward the subependymal zone of the optic tectum, curve along the subventricular zone, and capillarize to feed the subependymal capillary bed and the layers of the optic tectum (Figure 27). The strongest branch supplies the semicircular torus (Figure 27).

Within the transverse cleft between optic tecta and cerebellum, the caudal superior mesencephalic artery gives off caudally directed **cerebellar arteries** (Figures 27 and 31). Up to four cerebellar arteries per side may arise from the posterior superior mesencephalic arteries to supply the cerebellum via its rostral surface (Figure 31).

**Figure 30:**
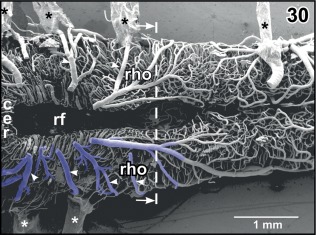
Microvascular anatomy of the cerebellar (cer)—rhombencephalic (rho) area. Dorsal view. Choroid plexus of the fourth ventricle is removed to expose the rhombencephalic fossa (rf). Note the circumferentially running tegmental veins (arrowheads) which drain into the choroid plexus of the fourth ventricle. Asterisks mark conductive bridges

**Figure 31:**
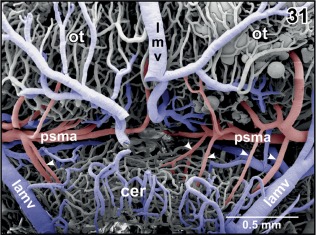
Arterial supply and venous drainage of the cerebellum. Rostral is at top. Choroid plexus IV is removed to expose the course of the posterior superior mesencephalic artery (psma) within the cleft between cerebellum (cer) and optic tecta (ot). Note several small cerebellar arteries (arrowheads) branching off the posterior superior mesencephalic artery

The **basilar artery** forms by the junction of right and left posterior branches of the cerebral arteries. It runs along the ventral midline of the rhombencephalic tegmentum and the medulla oblongata toward caudal (Figures 1, 3, and 5). In its rostral portion, it bilaterally issues the **auditory arteries**. Sites of origin of right and left auditory arteries may be right opposite to each other or may be shifted. Occasionally, one auditory artery may even branch off the posterior branch of the cerebral carotid artery (Figure 3). Three to four millimeters posterior to its rostral beginning the basilar artery communicates via the **communicating arteries with the vertebral arteries** (Figure 3). Like auditory arteries, communicating arteries leave the basilar artery at the same level (Figure 3) or are shifted to a varying degree. In few cases, communicating arteries bifurcate shortly before they join the basilar artery at both (Figure 3, inset a) or at one side only (Figure 3, inset b). Posterior to the origin of the communicating arteries with the vertebral arteries, the basilar artery continues as **ventral spinal artery**. This artery runs in the ventral spinal fissure toward the posterior end of the spinal cord.

**Figure 32:**
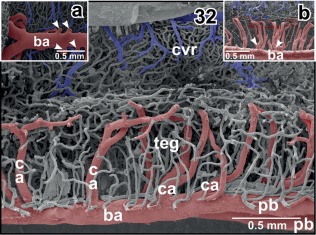
Vascular architecture of the rhombencephalic tegmentum (teg) and cochlear‐vestibular nuclear regions (cvr). Slightly parasaggitally sectioned rhombencephalon. Rostral is to the right. Note origin, course and branching patterns of central arteries (ca). **Inset a**. Central arteries (arrowheads) branching off the lateral circumference of the basilar artery (ba). **Inset b**. Central arteries branching immediately after their origin from the basilar artery (arrowheads)

**Figure 33:**
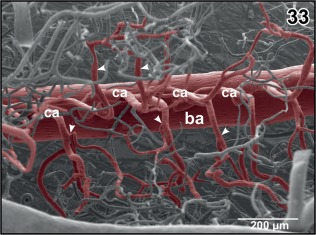
Branching patterns of central arteries. Dorsal view at bifurcating central arteries (ca) after removal of subependymal vessels. Note branches to right or left tegmental areas (arrowheads)

**Figure 34:**
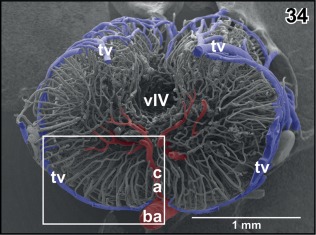
Microvascular anatomy of the caudal rhombencephalon. Transverse section at the level indicated by the dashed line in Figure 30. Frontal view indicated by arrows in Figure 30. Note an ascending central artery (ca) supplying the right side and horizontally running branches of a more anterior central artery on the left side of the rhombencephalon

**Figure 35:**
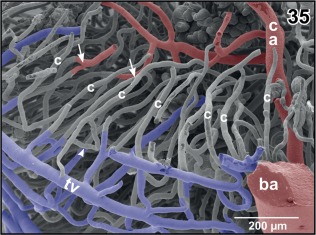
Radial arrangement of rhombencephalic tegmental capillaries (c). Detail from Figure 34 (box). Specimen slightly tilted. Note H‐shaped (arrowhead) and Y‐shaped (arrows) branchings

Basilar artery and ventral spinal artery give off **central arteries**. In the anterior portion of the basilar artery central arteries either ascend from the dorsal aspect of the basilar artery (Figure 32) or branch off the lateral surface to then bend over to the dorsal side (Figure 32, inset a). Central arteries either ascend vertically or slightly inclined toward rostral (Figures 32 and 33). Close beneath the central canal, they bifurcate into a left and right branch which curve around the central canal to ascend further dorsally to supply the dorsal spinal cord areas (Figure 34). Patterns of origin vary greatly. Central arteries may originate as an individual vessel or two to three central arteries may share a common stem and split into individual vessels at different levels within the ventral spinal cord fissure (Figure 32, inset b). Capillaries run in a radiating manner centrifugally toward the pial surface where they drain into circumferentially running venules (Figure 35).

### 3.2 Gross venous drainage

Course, calibers, and branching patterns of right and left cerebral drainage routes may greatly vary. In detail, olfactory bulbs, accessory olfactory bulbs, telencephalon, and rostral thalamic areas drain via prominent **lateral telencephalic veins** and a single **interhemispheric vein** (Figures 12, 15, and 16).

**Figure 36:**
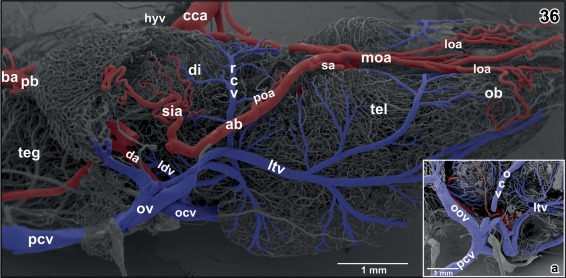
Gross arterial supply and venous drainage of olfactory bulb (ob), telencephalon (tel) and diencephalon (di). Ventro‐lateral view. Rostral is to the right. Note the lateral telencephalic vein (ltv) and its dorsal and ventral tributaries. **Inset a**. Confluence of cerebral veins at the level of the prootic foramen. Lateral view

**Figure 37:**
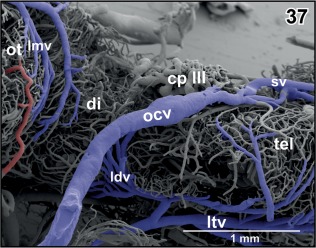
Course and caliber of the (right) oblique cranial vein (ocv). Lateral view at the optic tectum (ot), diencephalon (di), and caudal telencephalon (tel). Rostral is to the right

**Sagittal veins** primarily drain the meninges overlaying the dorsal telencephalic hemispheres. They drain into the rostromedial tip of the oblique cranial veins (Figures 17, 19, and 21). The **oblique cranial veins** extend in a caudalwards open V shape along the dorsal border between caudal poles of telencephalic hemispheres and adjacent epithalamic‐dorsal thalamic border regions toward caudolateral (Figures 19, 21, 36, and 37). They also drain part of caudal pallial areas, the paraphysis, and the choroid plexus of the third ventricle via the **choroidal vein** (Figures 17 and 36).

The **lateral telencephalic vein** shows a fan‐like pattern and forms from several large branches. It takes a caudoventral course along the lateral surface of the telencephalic hemisphere and drains into the **ophthalmic vein** (Figure 36 and inset a). Branches of the lateral telencephalic vein embrace dorsal, lateral, and ventral olfactory bulb, accessory olfactory bulb areas, and medial, dorsal, lateral, and ventral pallial and striatal areas.

**Figure 38:**
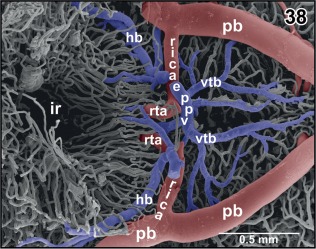
Anatomy of the encephalo‐posthypophysial portal system. Ventral aspect. Hypophysis and caudal infundibulum are removed. Note the hypothalamic branch (hb) and the ventral tegmental branches (vtb) of the portal system

**Figure 39:**
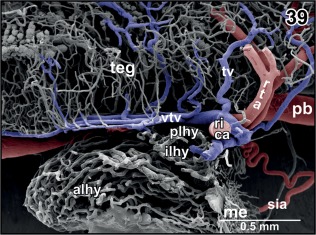
Vascular anatomy of the hypophysis. Longitudinal section through mesencephalic tegmentum and hypophysis. Note the drainage of the encephalo‐posthypophysial portal system into the rostro‐dorsal area of the posterior lobe of the hypophysis (plhy)

The **retrochiasmatic vein** which drains ventral hypothalamic areas (Figures 1, 2, 4, 5, and 36) joins the lateral diencephalic vein to jointly empty into the lateral telencephalic vein (Figure 36).

The main trunk of the **interhemispheric vein** ascends in the interhemispheric fissure in a slight concave curve from the rostro‐basal telencephalon toward the wedge‐like dorso‐medial border region of the telencephalic hemispheres and the diencephalic epithalamus (Figures 15 and 16). Its rostral branches, drain ventro‐caudal olfactory bulb, striatal, and septal areas. Caudally directed branches drain the vascular organ of the terminal lamina and part of the rostral preoptic area (Figure 15). The interhemispheric vein drains into the choroid plexus of the third ventricle (Figure 15, inset a).

The diencephalon bilaterally drains via several routes. These are (a) the lateral diencephalic vein (see above), (b) the single longitudinal mesencephalic vein, (c) the retrochiasmatic vein, and (d) the hypothalamic branch of the encephalo‐posthypophysial portal vein.

The **longitudinal mesencephalic vein** is a very conspicuous vein. Its main trunk runs dorso‐medially between the optic lobes of the mesencephalon and drains into the rostral area of the choroid plexus of the fourth ventricle. It forms from several branches which drain caudal epithalamic and thalamic areas, optic lobes, and latero‐dorsal regions of the mesencephalic tegmentum (Figures 18, 21, and 26).

The **retrochiasmatic vein** drains rostro‐ventral regions (post‐ and suprachiasmatic regions) of the infundibular lobe (Figures 1, 2, 4, 5, 18, and 36). It empties into the lateral diencephalic vein (see above) (Figure 36).

The **hypothalamic branch of the encephalo‐posthypophysial portal vein** drains the caudal infundibular lobe, the dorsal hypothalamus, and adjacent areas of the ventral thalamus (Figures 5, 6, 7 and 38). It runs adjacent to the rostral aspect of the anterior portion of the posterior branch of the cerebral carotid artery toward the retroinfundibular communicating artery. It follows this artery toward the midline and then descends to drain into the dorsal side of the posterior lobe of the hypophysis (Figures 5, 28, 38, and 39). Right and left branches either drain individually or one or both branches join the **ventral mesencephalic tegmental vein(s)** to then drain into the posterior (neural) lobe of the hypophysis (Figures 5, 6, 28, and 39). Posterior, intermediate, and distal lobes of the hypophysis finally drain via the hypophyseal vein into the lateral telencephalic vein (Figures 1 and 36).

**Figure 40:**
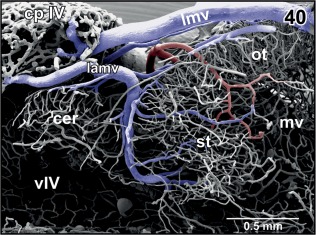
Venous drainage of the semicircular torus (st) into the anterior region of the choroid plexus of the fourth ventricle (cp IV). Medial aspect. Rostral is to the right

**Figure 41:**
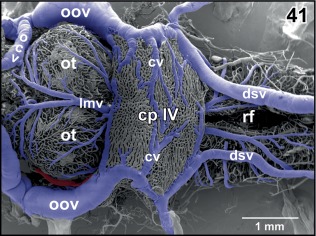
Vascular anatomy of the roof (tela choroidea) of the fourth ventricle. Dorsal aspect. Note that the choroid plexus (cp IV) does not cover the entire length of the rhombencephalic fossa (rf)

The mesencephalic optic lobes drain via the single **longitudinal mesencephalic vein** and **lateral and caudal mesencephalic veins** into the choroid plexus IV (Figures 18, 19, 26, 40, and 41). Mesencephalic tegmental areas drain via two routes, namely (a) via **lateral tegmental veins** which take their origin at the ventral midline (Figures 3, 35, and 43), ascend along the lateral tegmental surface to finally drain into the choroid plexus IV (Figure 18) and (b) via **ventral mesencephalic tegmental veins** (Figures 5, 24, 28, 38, and 39).

The cerebellum drains via longitudinal mesencephalic vein and lateral mesencephalic veins into the choroid plexus of the fourth ventricle (Figures 31 and 40). The rhombencephalic tegmentum drains via **lateral rhombencephalic veins** which take their origin at the ventral midline (Figure 43). Veins ascend around the lateral tegmental surface and join with neighboring veins to form larger trunks which further ascend toward the margins of the rhombencephalic fossa (Figures 18 and 30). In the rostral area, they directly drain into the choroid plexus of the fourth ventricle via short ascending veins, in the caudal regions they mostly drain into a vein which runs along the dorsal margin of the rhombencephalic fossa toward rostral (Figures 30 and 41). This vein then also drains into the choroid plexus of the fourth ventricle.

**Figure 42:**
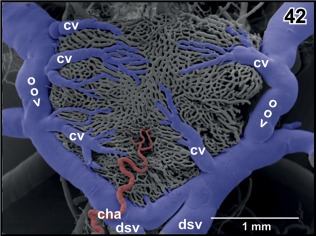
Arterial supply of the choroid plexus of the fourth ventricle by a small choroidal artery (cha). Note choroidal veins (cv) draining into the oblique occipital veins (oov)

The choroid plexus topping the rhombencephalic fossa varies interindividually in respect to shape, size, and vascular patterns (Figures 8, 9, 21, 41, and 42). In most cases, it has a shape like an isosceles triangle with the tip pointing caudally (Figures 21). In general, the plexus covers the entire (Figure 21), but in rare cases, it covers only part of the rhombencephalic fossa (Figure 41). The plexus forms from transversely orientated sheet‐like vascular lamellae (Figures 44 and 45). These lamellae consist of interconnected wide sinusoids with a somewhat thicker vessel at the ventral margins (Figure 45). In the rostral areas, lamellae are slightly inclined toward rostrally in the caudal portion they incline toward caudally (Figures 8 and 9). The tela of the choroid plexus IV shows a dense network of sinusoids of different shapes and sizes (Figures 21, 41, and 42). Several **choroidal veins** drain the plexus into the **oblique occipital veins** which run aside the lateral margins of the plexus toward rostro‐laterally (Figures 21, 41, and 42). In most cases, the oblique occipital veins form by the bifurcation of the **dorsal spinal vein** at the caudal tip of the plexus and empty into the **ophthalmic veins** (Figure 21). In a few cases, the dorsal spinal vein bifurcates more caudally and the two branches approach the caudal margin of the choroid plexus more laterally (Figure 41).

**Figure 43:**
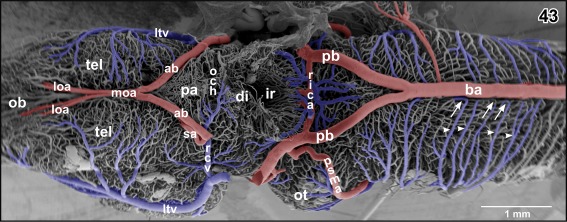
Major arteries and veins at the ventral surface of the brain of adult *X. laevis*. Ventral hypothalamus, hypophysis, and proximal portions of anterior and posterior branches of the cerebral carotid arteries are removed. Note the origin of the circumferential tegmental veins (arrowheads) at the ventral midline (arrows)

**Figure 44:**
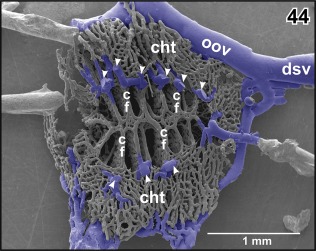
Vascular anatomy of the choroid plexus of the fourth ventricle. Ventral aspect. Note marginal choroidal tela (cht), central choroidal folds (cf), and left oblique occipital vein (oov). Arrowheads point at the sites where rhombencephalic tegmental veins drain into the choroid plexus

**Figure 45:**
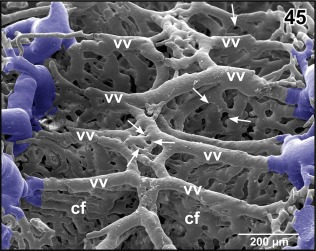
Detail from Figure 44. Vertical, transversely orientated choroidal folds (cf) made up from a dense 2D network of sinusoids. Choroidal folds reveal ventral venules (vv) which interconnect at the midline. Note the many sites of ongoing intussusceptive angiogenesis (arrows)

### 3.3 Microvascular patterns

**Figure 46:**
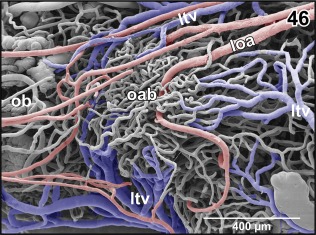
Microvascular anatomy of the accessory olfactory bulb (aob). Ventro‐lateral view. Note the dense capillary bed supplied by a branch of the lateral olfactory artery (loa) and the drainage by ventral, lateral, and dorsal branches of the lateral telencephalic vein (ltv)

**Figure 47:**
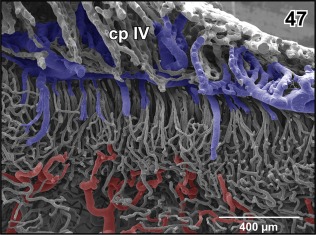
Microvascular pattern of the cochlear‐vestibular complex as seen from the rhombencephalic fossa. By the parasagittal section part of the subependymal capillary bed is sectioned‐off and underlying supplying arterioles are seen. Note that several capillaries join into single venules which ascend parallel to drain into the choroid plexus of the fourth ventricle (cp IV)

Most brain areas reveal a rather wide meshed 3D capillary network with a clear centrifugal arrangement. Few areas only display a dense capillary bed. These areas are in particular (a) the accessory olfactory bulb and (b) the vestibulo‐cochlear nucleus. The accessory olfactory bulb capillary bed is fed by branches of the lateral olfactory artery and drains into branches of the lateral telencephalic vein (Figure 46). The vestibulo‐cochlear capillary bed is the densest capillarized area found in the brain. Capillaries run parallel to each other over a short distance and are very closely spaced. They drain via small venules into the choroid plexus of the fourth ventricle (Figure 47).

## 4 DISCUSSION

Millard (1940) described the vascular anatomy of *X. laevis*. In her impressive study, she performs careful dissections and follows origin, course, and areas of supply of main cerebral vessels. Due to the limited depth of focus of the stereomicroscope, her study gives no information about the microvascular territories. SEM of vascular corrosion casts (Aharinejad & Lametschwandtner, 1992; Lametschwandtner, Lametschwandtner, & Weiger, 1990; Motta, Murakami, & Fujita, 1992; Murakami, 1971) overcame this limitation and for the first time, the 3D microvascular anatomy of the anuran (*B. bufo*; Albrecht et al., 1978; Lametschwandtner & Simonsberger, 1975; Lametschwandtner et al., 1976, 1977a, 1977b, 1977c, 1978, 1979a, 1979b, 1980a, 1980b), the urodelian brain (*T. cristatus and T. carnifex*; Lazzari et al., 1991), and *A. mexicanum* (Lazzari & Franceschini, 2003) was shown. The spatial resolution of the SEM is sufficiently high to clearly differentiate arteries from veins by means of the characteristic endothelial cell nuclei imprint patterns displayed on the surfaces of cast vessels (Miodonski et al., 1976; see also Figure 16, insets a and b) and enables to demonstrate origin, caliber, course, and branching patterns of individual blood vessels throughout the whole organ. If this technique is further supplemented by corresponding tissue sections with subsequent histomorphological analyses areas of supply and drainage of individual blood vessels can be defined with high reliability. Sectioning of vascular corrosion casts and/or removal of vessels layer by layer by using fine‐tipped insects pins allows further insights into individual microvascular patterns of topographically clearly defined brain areas.

If we compare our present findings in adult *X. laevis* with those gained earlier using the same technique in adult *B. bufo* (Albrecht et al., 1978; Lametschwandtner & Simonsberger, 1975; Lametschwandtner et al., 1976, 1977a, 1977b, 1977c, 1978, 1979a, 1979b, 1980b) and with those from a series of carefully performed early LM studies on the brain vasculature of adult anurans (Abbie, 1934; Craigie, 1938; Dierickx et al., 1970, 1974, 1971; Gaupp, 1899; Gillilan, 1967; Millard, 1940; Rex, 1893; Roofe, 1935; Schöbl, 1882; Socha, 1930), we find that the pipid frog *Xenopus* owns a greater intra‐ and interindividual variation in vessel origins, calibers, and courses than reported in Bufonidae and Ranidae. This has to be questioned, as a comparison of results gained by such different techniques as SEM of vascular corrosion casts and stereomicroscopic analyses of Indi‐ink or India‐ink/gelatin injected brains with or without prior clearing according to Spalteholz (1914) is problematic. With the latter technique, it is difficult to follow vessel courses over longer distances, particularly if vessels frequently change levels and overlay each other. This may lead to an erroneous interpretation of courses and branching patterns within the limited depth of focus of the stereomicroscope as compared to the high depth of focus of the SEM. Perfusion of the vascular system with, for example, the lipophilic dye DiI analyzed by confocal fluorescent microscope imaging (Hughes et al., 2014; Saltman, Barakat, Bryant, Brodovskaya, & Whited, 2017) overcomes many of these difficulties. However, it still is difficult to expose individual vessels in both, India‐ink or DiI injected specimens over longer distances without destroying vessels, surrounding tissues or anatomical landmarks.

A comparison of the vascular architecture of the brain parenchyma of urodelians (*T. cristatus and T. carnifex*, *A. mexicanum*) gained by SEM of vascular corrosion casts (Lazzari et al., 1991; Lazzari & Franceschini, 2003) with that of anurans shows convincingly that urodelians supply the brain parenchyma by hairpin‐like vascular loops which arise from the leptomeningeal surface arteries in an acute angle, extend into varying depths of the brain parenchyma before they return in close contact with each other to drain into meningeal veins. Capillary loops are described either as variously bent, inclined or twisted (Lazzari et al., 1991). This pattern contrasts to the anuran pattern where parenchymal vessels form a 3D network, which by its frequent interconnections, clearly enables a better blood supply to the brain parenchyma. Neither in adult *B. bufo* nor in adult *X. laevis*, hairpin‐like capillary loops are found. Instead, capillaries form a subependymal capillary network which in vascular corrosion casts clearly outlines the contours of brain ventricles.

According to Abbie (1934), the internal carotid artery in *R. temporaria* divides into cranial and caudal divisions. The anterior division (ramus anterior of Gaupp, 1899) divides at the anterior border of the optic tract into medial and lateral olfactory arteries. Confusingly, a few lines later, Abbie (1934) states that this artery "…takes its origin from one of the arteries to the diencephalon (see his fig. 3). The lateral olfactory artery courses along the telencephalic hemispheres toward rostral. It gives off many fairly large branches to the hemisphere (Abbie, 1934). In *R. pipiens*, *Rana clamitans*, and *R. catesbeiana*, Gillilan (1967) describes also a lateral olfactory artery which gives off a lateral striatal artery and continues as posterior telencephalic artery to supply dorsomedial areas of the telencephalon, epiphysis and thalamus. Interestingly, Craigie (1938) in his comprehensive study on the blood vessels of the brain substance in some amphibians does not describe a lateral olfactory artery in *R. pipiens*. In *Rana esculenta*, Socha (1930) does not find a lateral olfactory artery or an artery which takes a similar course. He, however, describes an artery, which ascends between the telencephalic hemispheres and supplies by small branches telencephalic areas. This artery most probably is the ramus hemispherii medialis dorsalis described in *R. temporaria* by Dierickx et al. (1974), in *B. bufo* by Albrecht et al. (1978), Lametschwandtner and Simonsberger (1975), Lametschwandtner et al. (1976, 1977a, 1977b, 1977c, 1979a, 1979b, 1980) and in *X. laevis* (this study).

In respect to the lateral olfactory artery described by Abbie (1934) in *R. temporaria* and by Gillilan (1967) in *R. pipiens*, *R. clamitans*, and *R. catesbeiana*, but not described by Socha (1930) in *R. temporaria* and by Craigie (1938) in *R. pipiens*, it is interesting to compare course and pattern of this artery (shown in Abbie's fig. 3) with that of the lateral telencephalic vein found in *X. laevis* (this study, see Figures 12 and 36). To finally clarify the discrepancies in respect to the presence or absence of this artery in ranid species a SEM analysis of brain vascular corrosion casts of the ranid species cited needs to be done.

In his comprehensive study on the blood vessels of the brain substance in some amphibians, Craigie (1938) mentions that from the transverse anastomotic channel (retroinfundibular communicating artery; Cruz, 1959) “…a pair of relatively large arteries run directly dorsal within the brain tissue and branch to supply much of the midbrain and the caudal diencephalon.” Our SEM analyses of vascular corrosion casts of the brain of adult *X. laevis* confirm the presence of these vessels and—due to the advantages of SEM of vascular corrosion casts over binocular dissections or LM analyses of India‐ink injected, cleared, and sectioned brain tissue—demonstrates precisely and reliably course, branching pattern, and areas of supply of these vessels.

In adult *Xenopus* (present study), a closed arterial circle of Willis was found in about two‐third of the specimens. In these cases, the circle formed by the anterior and posterior branches of right and left cerebral carotid arteries whereby the latter joined the ventro‐medially located basilar artery at the level of the cerebellum (see Figures 1, 2, and 5). In cases of an open arterial circle of Willis, the anterior branches of right and left cerebral arteries did not join at the caudo‐ventral interhemispheric fissure. Interestingly, in rare cases, a continuous retroinfundibular communicating artery which interconnected right and left posterior branches at the level of the hypophysis was lacking, but right and left stems of this vessel bent close to the midline toward dorsal and continued as tegmental arteries with only a thin interconnecting vessel to the opposite site or with no interconnection at all (Figures 6 and 7).

For a long time, the arterial circle of Willis which was reported to be complete in only 21% of humans studied so far (Lippert & Pabst, 1985) was solely considered to be a compensatory mechanism which in cases of occlusion or stenosis of an internal carotid artery or a vertebral artery enables the redistribution of blood flow (Nornes, 1973). Recently, however, the arterial circle of Willis was considered from an evolutionary point of view and an additional function, namely a function as a passive pressure dissipating system which protects cerebral arteries and the blood‐brain‐barrier from hemodynamic stress was postulated (Vrselja, Brkic, Mrdenovic, Radic, & Curic, 2014). In men, the arterial circle of Willis comprises anterior and posterior communicating arteries which have much thinner calibers (≤0.3 mm) than the internal carotid arteries or basilar artery, whereas the arteries forming the arterial circle in *Xenopus* have rather similar calibers (see Figures 1 and 5). It therefore remains open if the arterial circle of Willis in *Xenopus* serves the same two functions as that in men where additionally much higher blood pressures in combination with the rigid cranial cavity exert a much higher pulsatile stress to brain vessels (Vrselja et al., 2014). If the higher prevalence of a closed arterial circle of Willis in *X. laevis*, a species which is secondary aquatic (i.e., derives from a terrestrial ancestor species), is the heritage from the initial land‐living lifestyle or is simple due to the nature of its arterial circle cannot be answered yet. To do so, data on the arterial circle of Willis from other land‐living amphibians is needed.

*X. laevis* has a diencephalic and a rhombencephalic choroid plexus only; it lacks choroid plexuses in the telencephalic (lateral) ventricles. It is assumed that choroid plexuses participate to 80% in the formation of cerebrospinal fluid (CSF) by two means, namely by (a) passive filtration of fluid across the highly permeable capillary endothelium and (b) regulated secretion across the single‐layered choroidal epithelium (Brinker, Stopa, Morrison, & Klinge, 2014). In respect to (a) the high venous input—in comparison with the small arterial input found in both choroid plexuses of *X. laevis*—is remarkable. In studies on structure and function of choroid plexuses in mammals and man, which own two choroid plexuses in the lateral ventricles, and one each in the diencephalon and the rhombencephalon, an inflow via a choroidal artery and an outflow through venules is described (e.g., Meeker, Williams, Killebrew, & Hudson, 2012); an additional venous inflow from surrounding cerebral areas is not reported.

Interestingly, vascular casts of choroid plexuses III and IV show many holes of different shapes and sizes indicative of ongoing intussusceptive (nonsprouting) angiogenesis (Figures 23 and 45). Obviously, intussusceptive angiogenesis, a process by which preexisting vessels split or remodel through the formation of transluminal tissue pillars (Caduff, Fischer, & Burri, 1986; Diaz‐Flores et al., 2017; Mentzer & Konerding, 2014) occurs not only during tissue development or in some pathological processes including tumours (Diaz‐Flores et al., 2017; Mentzer & Konerding, 2014), but also in adult tissue. Intussusceptive angiogenesis by its facets (intussusceptive microvascular growth, intussusceptive arborisation, intussusceptive branching remodeling, intussusceptive pruning; Burri, Hlushchuk, & Donov, 2004) serves to adapt a preexisting vasculature to changing needs of tissues supply and waste removal. In case of the adult choroid plexuses with their high venous inflow, intussusceptive angiogenesis may serve to optimize vascular perfusion for CSF production.

## CONFLICT OF INTERESTS

The authors have no conflict of interests to declare.

## AUTHOR CONTRIBUTIONS

Alois Lametschwandtner performed resin injections, processing and SEM analyses of vascular corrosion casts, and tissues sections. Both Alois Lametschwandtner and Bernd Minnich equally participated in drafting, critical revision, and final approval of the manuscript and the figure plates.
